# Blockade of EGFR Activation Promotes TNF-Induced Lung Epithelial Cell Apoptosis and Pulmonary Injury

**DOI:** 10.3390/ijms20164021

**Published:** 2019-08-17

**Authors:** Toshimitsu Yamaoka, Satoru Arata, Mayumi Homma, Tetsuya Homma, Sojiro Kusumoto, Koichi Ando, Ryou Manabe, Yasunari Kishino, Motoi Ohba, Junji Tsurutani, Masafumi Takimoto, Tohru Ohmori, Hironori Sagara

**Affiliations:** 1 Advanced Cancer Translational Research Institute, Showa University, 1-5-8 Hatanodai, Shinagawa-ku, Tokyo 142-8555, Japan; 2 Division of Allergology and Respiratory Medicine, Department of Medicine, Showa University School of Medicine, Tokyo 142-8666, Japan; 3 Department of Biochemistry, Faculty of Arts and Sciences, Showa University, 4562 Kamiyoshida, Fujiyoshida, Yamanashi 403-0005, Japan; 4 Department of Pathology & Laboratory Medicine, Showa University School of Medicine, Tokyo 142-8555, Japan

**Keywords:** TNF, EGFR, apoptosis, lung injury, transactivation

## Abstract

Pneumonitis is the leading cause of death associated with the use of epidermal growth factor receptor (EGFR) tyrosine kinase inhibitors (EGFR-TKIs) against non-small cell lung cancer (NSCLC). However, the risk factors and the mechanism underlying this toxicity have not been elucidated. Tumor necrosis factor (TNF) has been reported to transactivate EGFR in pulmonary epithelial cells. Hence, we aimed to test the hypothesis that EGFR tyrosine kinase activity regulates TNF-mediated bronchial epithelial cell survival, and that inhibition of EGFR activity increases TNF-induced lung epithelial cell apoptosis. We used surfactant protein C (SPC)-TNF transgenic (tg) mice which overexpress TNF in the lungs. In this model, gefitinib, an EGFR-TKI, enhanced lung epithelial cell apoptosis and lymphocytic inflammation, indicating that EGFR tyrosine kinase prevents TNF-induced lung injury. Furthermore, IL-17A was significantly upregulated by gefitinib in SPC-TNF tg mice and p38MAPK activation was observed, indicative of a pathway involved in lung epithelial cell apoptosis. Moreover, in lung epithelial cells, BEAS-2B, TNF stimulated EGFR transactivation via the TNF-α-converting enzyme in a manner that requires heparin binding (HB)-EGF and transforming growth factor (TGF)-α. These novel findings have significant implications in understanding the role of EGFR in maintaining human bronchial epithelial cell homeostasis and in NSCLC treatment.

## 1. Introduction

Epidermal growth factor receptor (EGFR) tyrosine kinase inhibitors (TKIs) are given as standard therapy to non-small cell lung cancer (NSCLC) patients who carry activating mutations in the EGFR gene. The EGFR-TKI gefitinib, when administered to NSCLC patients carrying activating *EGFR* mutations (e.g., 15-bp deletion in exon 19, L858R in exon 21), significantly improved response and survival [1,2]. Furthermore, these EGFR mutations were predominantly found in patients of East-Asian origin [3]. However, although EGFR-TKI treatment has significant effects on response and prognosis, a study has reported that 40% of patients treated with EGFR-TKI experience severe adverse events, and treatment had to be discontinued in 7.7% of the patients because of unmanageable severe adverse events [4]. Diarrhea and rashes were the most frequent adverse effects but were well-tolerated and manageable. Toxic deaths were rare at 1.7%, and pneumonitis emerged as the most common severe form of toxicity as it accounted for more than half the toxic deaths following the administration of EGFR-TKIs [4]. Based on a retrospective analysis in Japanese patients, the EGFR-TKI treatment-related risk of pneumonitis and mortality are 3.5% and 1.6%, respectively [5]. According to a worldwide meta-analysis, the risk for pneumonitis is at 1.7% and the mortality risk is at 0.5% following EGFR-TKI treatment. The incidence rates for EGFR-TKI treatment-related toxicity are higher among the Japanese than in other races, but the reason why Japanese NSCLC patients are susceptible to EGFR-TKI-induced pneumonitis is not clear. Notably, the incidence of pneumonitis was higher in male patients with smoking history, pre-existing interstitial pneumonitis, and poor performance status, which are considered as grade 3 or 4 by the Eastern Cooperative Oncology Group [3]. However, the biological characteristics associated with EGFR-TKI-induced pneumonitis are yet to be determined.

EGFR is a receptor tyrosine kinase belonging to the ErbB receptor family. It regulates multiple aspects of pulmonary epithelial cell homeostasis in response to injury, including cell proliferation, cell survival, barrier function, and ion transport [6,7]. EGFR and other ErbB family members can be activated by direct interaction with EGF-like ligands. This initiates receptor dimerization and increased kinase activity, which leads to the activation of downstream signaling factors, such as phosphatidylinositol 3-kinase (PI3K)/AKT and mitogen activated protein kinase/extracellular-signal-regulated kinase (MAPK/ERK) pathways [8]. In addition, EGFR can be transactivated by various extracellular stimuli, such as antagonists of G protein-coupled receptors (GPCR) and of cytokine receptors [9]. Tumor necrosis factor (TNF), interleukin (IL)-1β, IL-8, IL-13, and interferon (IFN)-γ have been reported to transactivate EGFR in pulmonary epithelial cells [10,11,12,13].

Tumor necrosis factor is a potent pro-inflammatory cytokine that regulates diverse biological process including cell survival, apoptosis, proliferation, and migration in various kinds of tissues. Dysregulation of TNF is implicated in numerous disease states such as rheumatoid arthritis, Crohn’s disease, ulcerative colitis, and cancer [14]. Furthermore, neutralizing antibodies for TNF have been developed as therapeutics for these autoimmune inflammatory diseases. TNF regulates both anti- and pro-apoptotic signal transduction pathways and maintains balance between these two pathways. TNF-induced anti-apoptotic pathways include PI3K/AKT, ERK/MAPK, and nuclear factor kappa-light-chain-enhancer of activated B cells (NFκB), whereas pro-apoptotic TNF induced-pathways include p38 MAPK and stress-activated protein kinase/c-Jun NH2-terminal kinase (SAPK/JNK) [15,16,17]. During pneumonitis, inflammatory cytokines, such as TNF, IL-1β, and granulocyte-macrophage colony-stimulating factor (GM-CSF) are released, leading to cell apoptosis, tissue necrosis, and micro-vascular dysfunction [18].

Several EGFR ligands are cleaved from the cell surface by a disintegrin and metalloproteinase domain-containing protein 17 (ADAM17), also called TACE (TNF-α-converting enzyme), in a process termed ectodomain shedding [19]. TACE-mediated release of EGFR ligands from the cell surface via ectodomain shedding is considered essential for EGFR activation. 

Previously, we have reported that EGFR and human epithelial growth factor receptor 2 (HER2) transactivation by TNF promote the survival response of colon epithelial cells, and that TNF transactivation of EGFR/HER2 required Src-kinase activity with AKT as a downstream target [20]. These findings illustrate an important relationship between the TNF and EGFR signal transduction pathways and their role in epithelial cell survival in a cytokine-enriched environment during acute injury response. Therefore, we hypothesize that EGFR-TKI may enhance the development of lung injury in TNF-overexpressing lung tissue through blockade of TNF-induced EGFR transactivation. 

In this study, we employed surfactant protein C (SPC)-TNF transgenic (tg) mice, in which the *TNF-α* gene was placed under the control of the transcriptional promoter of the *SPC* gene. TNF was expressed in the lungs of these mice, producing alveolitis, alveolar disruption, and progressive pulmonary fibrosis [21]. Our results demonstrate that the administration of an EGFR-TKI, gefitinib, remarkably enhanced lung inflammation in SPC-TNF tg mice, and significantly induced apoptosis of pulmonary cells via the p38 MAPK pathway. Human lung epithelial cells, BEAS-2B, exhibited EGFR transactivation by TNF via cleavage of heparin-binding (HB)-EGF by TACE and was thereby protected from TNF-induced apoptosis. Although EGFR-TKIs are promising therapeutics for *EGFR* mutation-positive patients, it should be carefully administered to patients with accompanying COPD, pneumonitis, and pneumonia, and enrichment of inflammatory cytokines.

## 2. Results

### 2.1. Gefitinib Treatment Enhanced Lymphocytic Infiltration and Lung Inflammation in SPC-TNF Transgenic Mice

EGFR regulates multiple epithelial responses to injury; however, the role of EGFR activity in immune-mediated lung injury has not been examined. To address the hypothesis that EGFR tyrosine kinase activity protects lung tissue from inflammatory cytokine storms, we used SPC-TNF transgenic mice, which had increased expression of TNF in type II epithelial cells (Table 1). These mice have lymphocytic and fibrosing alveolitis, which resemble many aspects of human severe emphysema and pulmonary fibrosis [21]. 

**Figure 1:**
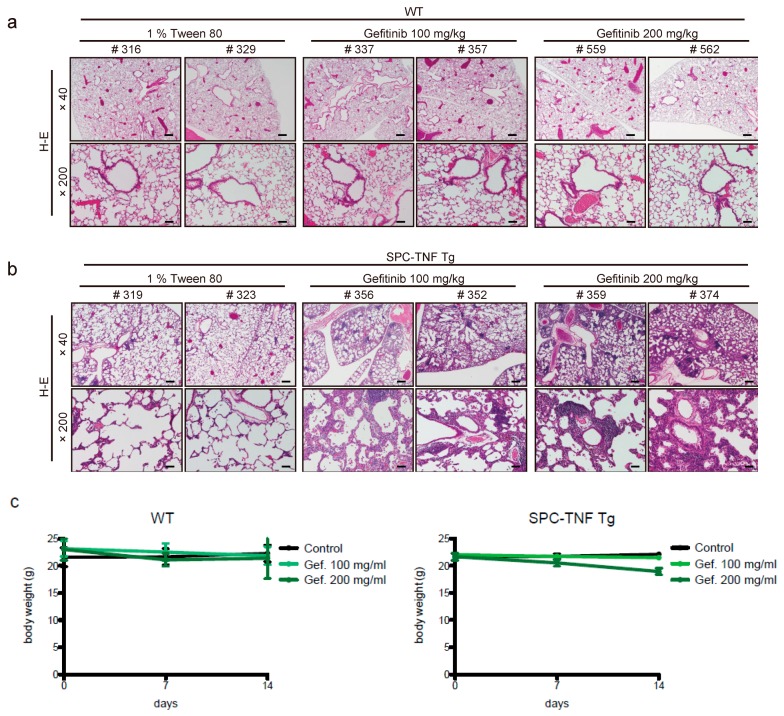
Gefitinib enhances lymphocyte infiltration and causes lung tissue inflammation. Lung tissue sections of (**a**) wild type (WT) mice and (**b**) surfactant protein C-tumor necrosis factor (SPC-TNF) transgenic (tg) mice were stained with hematoxylin and eosin. The images were obtained at a magnification of 40× and 200×, in the upper and lower rows, respectively. (**c**) Body weight changes are shown for WT and SPC-TNF Tg mice over 14 days of daily oral treatment with 1% tween 80 as control or with gefitinb (100 or 200 mg/kg). The WT; C57BL/6 or SPC-TNF tg mice used in these studies were 6-8 weeks old. The scale bars represent 500 μm and 100 μm for 40× and 200× magnification, respectively. Data in the figure represent mean body weights ± SEMs; * *p* < 0.05, Student’s *t*-test.

Gefitinib (100 or 200 mg/kg) was orally administered once daily in wild type (WT; C57BL/6) or SPC-TNF tg mice for 14 days. In wild type (WT) mice, no significant pathological change was observed (Figure 1a). In SPC-TNF tg mice, administration of gefitinib resulted in severe lymphocytic infiltration in the alveolar septa, and the mice also developed thicker alveolar septa than non-administered mice (Figure 1b). After 14 days of administration, there was also no death related to gefitinib administration. Moreover, there was no change in body weight in either WT or SPC-TNF tg mice (Figure 1c). To determine the inflammatory cytokines that mediate lymphocytic infiltration in SPC-TNF tg mice, real-time RT PCR was performed (Table 2 and Table 3). In wt mice, although mRNA levels for the pro-inflammatory cytokine IL-1β were significantly increased, no inflammation and lung injury was observed, suggesting a maintenance of balance by other anti-inflammatory cytokines, or through an unknown mechanisms. The mRNA level of IL-17α was found to be significantly increased by gefitinib administration. Taken together, these results suggest that treatment with EGFR-TKI might cause lung inflammation in SPC-TNF tg mice that overexpress TNF in the lung.

### 2.2. Indocyanine Green (ICG)-Loaded Liposomes Accumulated in Whole Lungs of Gefitinib-Treated SPC-TNF tg Mice

**Figure 2:**
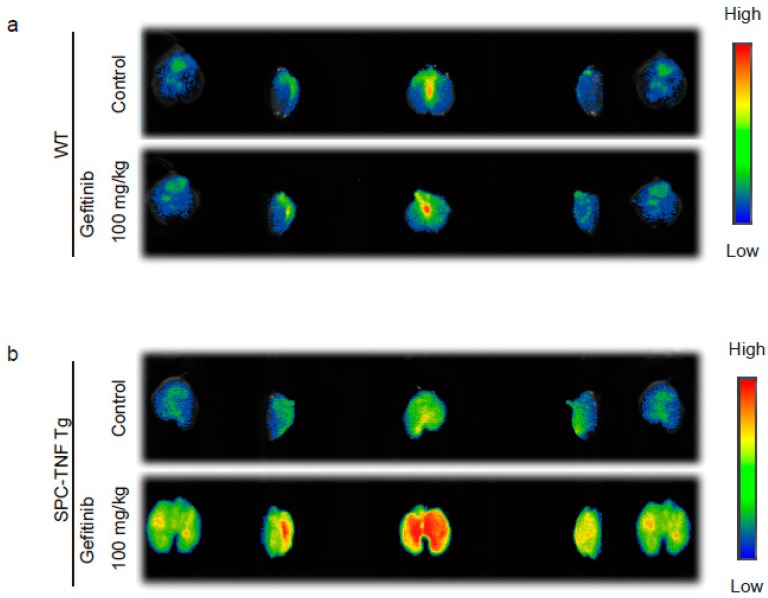
Gefitinib enhances lung inflammation in surfactant protein C-tumor necrosis factor (SPC-TNF) transgenic (tg) mice. Indocyanine green (ICG)-loaded liposomes carrying sialyl Lewis X were used to detect regions of inflammation in whole lungs of (**a**) wildtype (WT) and (**b**) SPC-TNF tg mice after daily gefitinib (100 mg/kg) treatment for 14 days. Images were captured using the Clairvivo OPT in vivo imaging system (Shimadzu, Kyoto, Japan).

To evaluate the extent of gefitinib-induced inflammation in mouse lungs, we used indocyanine green (ICG)-loaded liposomes, labeled with sialyl Lewis X (SLX) on the surface (SLX-Lipo-ICG). These SLX-Lipo-ICG could accumulate in the regions of inflammation or tumor owing to the interaction of SLX and E-selectin, which enhanced E-selectin expression by inflammatory cytokines, such as TNF, in vascular endothelial cells [22]. In WT and SPC-TNF tg mice, we measured whole-lung fluorescence ex vivo using the Clairvivo OPT fluorescence imaging system. We observed slight accumulation of ICG-loaded liposomes in whole lungs of SPC-TNF tg mice that were not treated with gefitinib compared with whole lungs of WT mice. Furthermore, after 14 days of gefitinib administration, accumulation of liposomes was enhanced in whole-lung samples taken from SPC-TNF tg mice. However, there was no enhancement in liposome accumulation after gefitinib treatment in WT mice (Figure 2a,b). These results suggest that EGFR tyrosine kinase activity protects mice from TNF-induced lung inflammation in mice model.

### 2.3. EGFR Tyrosine Kinase Activity Protected Lung Epithelial Cells from TNF-Induced Apoptosis in the SPC-TNF tg Mice Model

**Figure 3:**
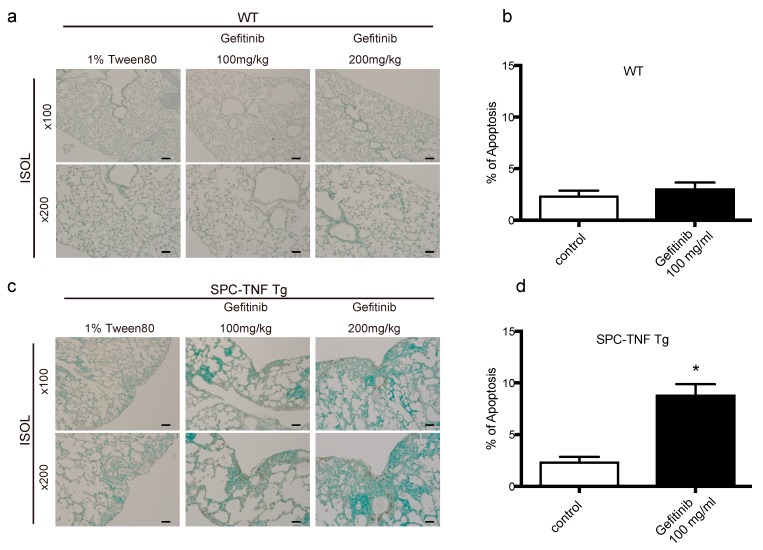

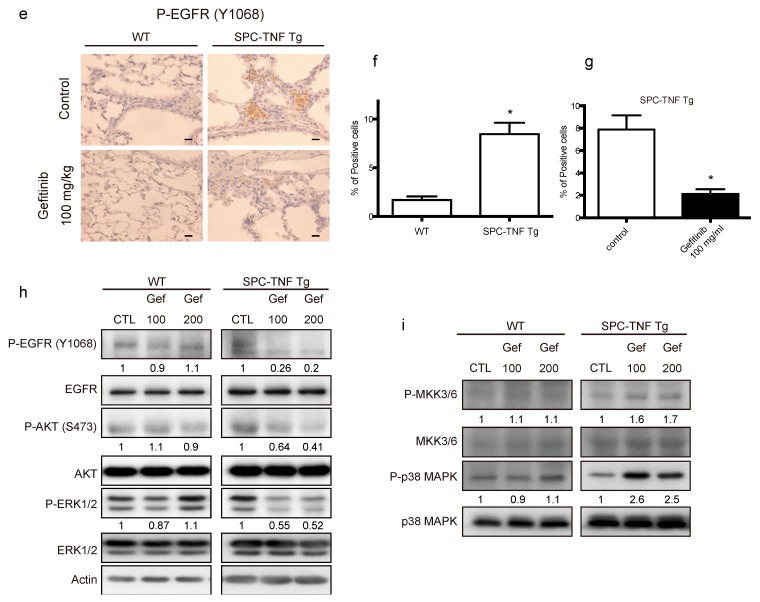
Gefitinib enhanced lung epithelial cell apoptosis via p38 mitogen-activated protein kinase (MAPK) pathway in surfactant protein C-tumor necrosis factor (SPC-TNF) transgenic (tg) mice. Wildtype (WT) and SPC-TNF tg mice were administered daily with gefitinib (100 or 200 mg/kg) or 1% tween 80 by oral gavage for 14 days. Paraffin-embedded lung tissues were studied for apoptosis using in situ oligo ligation (ISOL) staining, and apoptotic nuclei were labeled with peroxidase in (**a**) WT and (**c**) SPC-TNF tg mice. Percentage of apoptosis was determined for at least 500 cells in (**b**) WT and (**d**) SPC-TNF tg mice. * *p* < 0.05 compared with control, Student’s *t-*test. The scale bar represents 200 μm and 100 μm for 100× and 200× magnification, respectively. (**e**) Phosphorylated (P) epithelial growth factor receptor (P-EGFR; Y1068) was detected in paraffin-embedded lung tissues by immunostaining. The scale bar represents 50 μm for 400× magnification. (**f**,**g**) The percentages of positive cells are shown in the control lung tissues of WT and SPC-TNF tg (**f**), and in the SPC-TNF tg mice, where both a control and gefitinib were administered (**g**). * *p* < 0.05 compared with WT (**f**), or control (**g**), Student’s *t-*test. (**h**) Western blot analysis of EGFR/P-EGFR (Y1068), AKT/P-AKT, and extracellular-signal-regulated kinase (ERK)1/2/P-ERK1/2 in lung tissues of WT and SPC-TNF tg mice. β-actin was included as a loading control. (**i**) Western blot analysis of p38 mitogen-activated protein kinase (MAPK)/P-p38 MAPK and MAPK kinase (MKK) 3/6/P-MKK3/6 in lung tissues of WT and SPC-TNF tg mice. β-actin was included as a loading control. Band intensity of phosphorylated EGFR (P-EGFR), AKT (P-AKT), ERK1/2 (P-ERK1/2), MKK3/6 (P-MKK3/6), and p38MAPK (P-p38MAPK) was quantified and determined using ImageJ software (NIH). The relative ratios compared to the control are shown.

TNF is a pleiotropic cytokine that activates both anti- and pro-apoptotic signaling pathways. Cell survival is determined by the balance of these two pathways [20]. Therefore, we detected the cells undergoing apoptosis by in situ oligo-ligation (ISOL) in WT and SPC-TNF tg mice under gefitinib administration or not (Figure 3a,c). Gefitinib administration significantly enhanced apoptosis of lung epithelial cells in SPC-TNF tg mice but not in WT mice (Figure 3b,d). EGFR phosphorylation (P-EGFR) was significantly increased in SPC-TNF tg mice compared to WT mice (Figure 3e,f). Moreover, EGFR tyrosine kinase activity, as indicated by EGFR phosphorylation, was significantly inhibited in SPC-TNF tg mice after gefitinib administration (Figure 3e,g). Activation by phosphorylation of downstream signals AKT and ERK1/2 also decreased in SPC-TNF tg mice (Figure 3h). Anti-apoptotic responses can be mediated by AKT, ERK1/2, or Raf; else, p38MAPK is activated through MKK3/6 phosphorylation, leading to apoptosis [23,24]. Therefore, apoptosis in SPC-TNF tg mice may be induced by p38 MAPK pathways via MAP kinase kinase (MKK) 3/6, in addition to the inhibition of AKT and ERK1/2 activation with gefitinib exposure (Figure 3i).

### 2.4. TNF Stimulated EGFR Phosphorylation and Prevented Apoptosis in Lung Epithelial Cells

**Figure 4:**
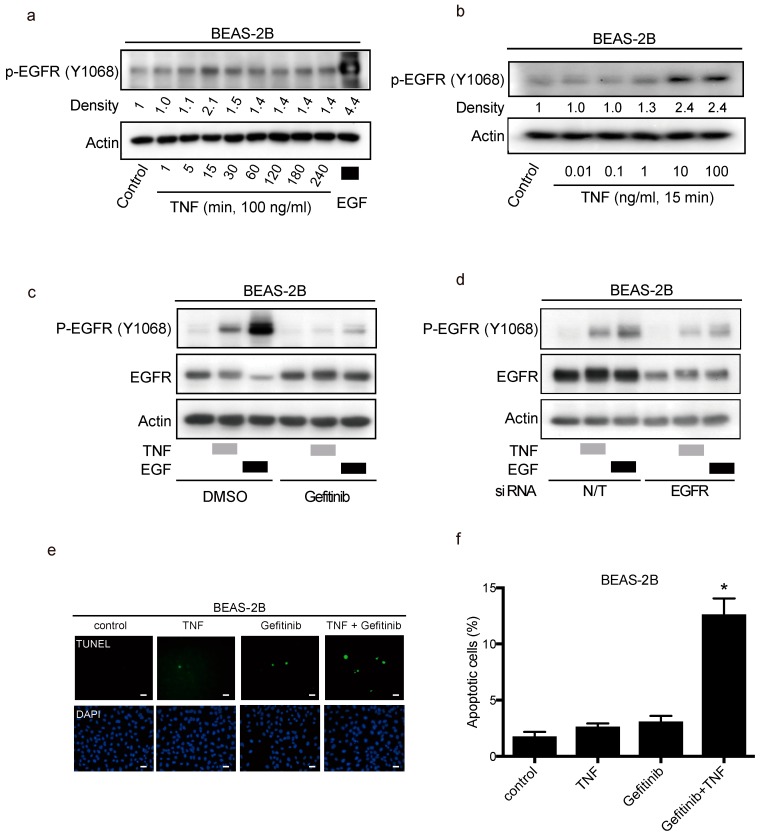
Transactivation of the epithelial growth factor receptor (EGFR) protects lung epithelial cells from apoptosis induced by tumor necrosis factor (TNF). Lung epithelial cells (BEAS-2B) were cultured and serum-starved for 16 h prior to treatment unless otherwise indicated (**a–e**). (**a–d**) BEAS-2B cell lysates were immunoblotted with the indicated primary antibodies after the following treatments, and β-actin was included as a loading control. (**a**) BEAS-2B cells were treated with TNF (100 ng/mL) for the indicated duration. Then the cells were treated with EGF (10 ng/mL) for 15 min as positive control. (**b**) BEAS-2B cells were treated with the indicated concentrations of TNF for 15 min. (**c**) BEAS-2B cells were treated with gefitinib (1 µM) for 1 h, and then treated with either EGF (10 ng/mL) or TNF (100 ng/mL) for 15 min. Band intensity of phosphorylated EGFR (P-EGFR) was quantified and determined using the ImageJ software (NIH). The relative ratios compared to the control are shown. (**d**) BEAS-2B cells were cultured on collagen-coated dishes, and transfected with either non-targeting (N/T) small interfering (siRNA) or with siRNA against EGFR for 48 h; cells were serum-starved for 16 h and then treated with EGF (10 ng/mL) or TNF (100 ng/mL) for 15 min. (**e**) BEAS-2B cells were treated with TNF (100 ng/mL) for 8 h in the presence or absence of gefitinib (1 µM). Cells were fixed for terminal deoxynucleotidyl transferase dUTP nick end labeling (TUNEL) assay, with apoptotic nuclei labeled with fluorescein isothiocyanate (FITC, green) and nuclei labeled with 4′,6-diaminido-2-phenylindole (DAPI, blue). FITC- and DAPI- labeled images were taken from the same field. The scale bar represents 100 μm. (**f**) Percentages of apoptotic cells were determined out of at least 500 cells. * *p* < 0.05, Student’s *t-*test, compared with the control, the treatment with TNF and with gefitinib.

To determine the mechanisms of EGFR transactivation by TNF exposure in lung epithelial cells, BEAS-2B cells were employed. At first, BEAS-2B cells were exposed to TNF (100 ng/mL) from 1 to 240 min; stimulation of EGFR phosphorylation was initiated from 15 min after TNF exposure (Figure 4a). Moreover, EGFR phosphorylation was enhanced by TNF in a dose-dependent matter (Figure 4b). TNF-induced EGFR phosphorylation was inhibited by gefitinib, suggesting the necessity of EGFR tyrosine kinase activity in this regard. (Figure 4c). Transfection of small interfering RNA (siRNA) against EGFR into BEAS-2B cells led to decreased EGFR expression and attenuated TNF-induced EGFR phosphorylation (Figure 4d). Therefore, TNF-induced EGFR phosphorylation required the expression and activation of EGFR. Interestingly, exposing BEAS-2B cells simultaneously with TNF and gefitinib, enhanced TNF-induced apoptosis (Figure 4e,f). These results suggest that EGFR phosphorylation by TNF inhibited TNF-induced apoptosis.

### 2.5. TNF Transactivates EGFR through TACE and Regulates Cell-Survival via the PI3-Kinase/AKT Pathway

**Figure 5:**
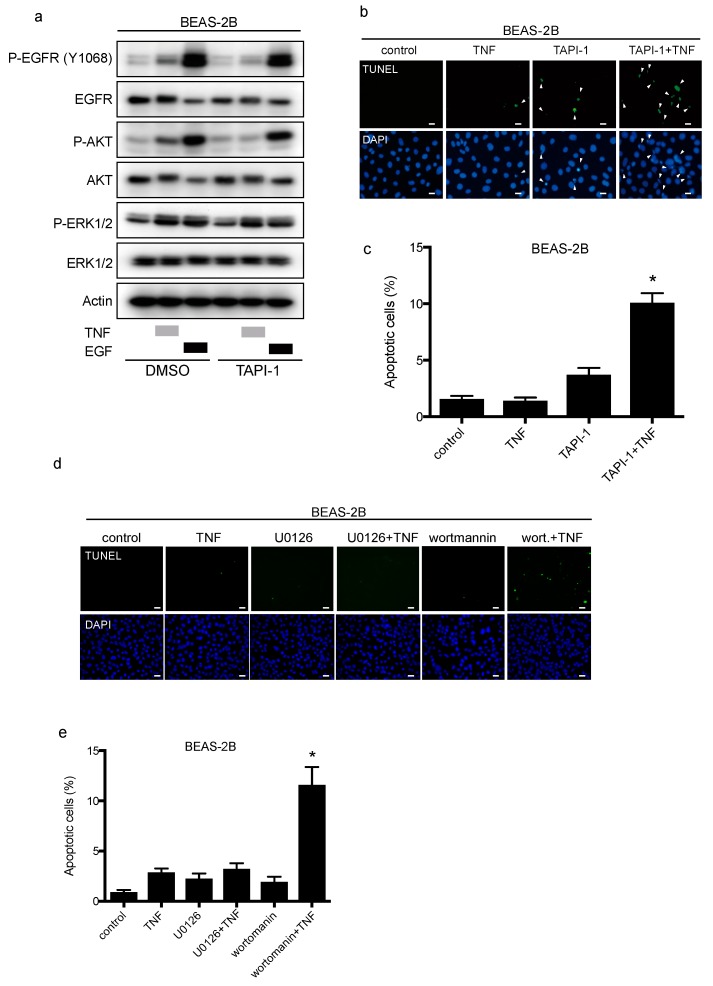
TAPI-1 enhances tumor necrosis factor (TNF)-induced lung epithelial cell apoptosis. Lung epithelial cells (BEAS-2B) were cultured and serum-starved for 16 h prior to treatment unless otherwise indicated (**a–d**). (**a**) BEAS-2B cells were treated with TAPI-1 (10 µM), a TNF-α converting enzyme (TACE) inhibitor, for 1 h, and then treated with either TNF (100 ng/mL) or epithelial growth factor (EGF, 10 ng/mL) for 15 min. Cell lysates were immunoblotted with the indicated primary antibodies. β-actin was included as a loading control. (**b**) BEAS-2B cells were treated with TNF (100 ng/mL) for 8 h in the presence or absence of TAPI-1 (10 µM). Cells were fixed for terminal deoxynucleotidyl transferase dUTP nick end labeling (TUNEL) assay, with apoptotic nuclei labeled with fluorescein isothiocyanate (FITC, green) and nuclei labeled with 4′,6-diaminido-2-phenylindole (DAPI, blue). FITC- and DAPI- labeled images were taken from the same field. The scale bar represents 50 μm. (**c**) Percentages of apoptotic cells were determined out of at least 500 cells. * *p* < 0.05, Student’s *t*-test, compared with the control, the treatment with TNF or TAPI-1. (**d**) BEAS-2B cells were treated with TNF (100 ng/mL) for 8 h in the presence or absence of a MAPK/ERK kinase (MEK) inhibitor, U0126 (10 µM), or a PI3 kinase (P13K) inhibitor, wortmannin (1 µM). Cells were fixed for TUNEL assay, with apoptotic nuclei labeled with FITC (green) and nuclei labeled with DAPI (blue). FITC- and DAPI- labeled images were taken from the same field. The scale bar represents 100 μm. (**e**) Percentages of apoptotic cells were determined out of at least 500 cells. * *p* < 0.05 compared with the control, the treatment with TNF or with wortmannin or U0126.

TACE facilitates the shedding of EGFR ligands, which are then available to bind and stimulate EGFR again in a process called the autocrine loop [6]. In BEAS-2B cells, the TACE inhibitor TAPI-1 suppressed TNF-induced EGFR phosphorylation, but not EGF-stimulation (Figure 5a). Furthermore, TAPI-1 enhanced TNF-induced apoptosis significantly (Figure 5b and c), suggesting that TACE mediates EGFR phosphorylation, leading to cell survival upon TNF exposure. The major downstream signals from EGFR include the PI-3K/AKT and RAS/ERK pathways. TNF transactivated EGFR by shedding mainly two EGFR ligands, and then we determined the signaling molecules downstream of EGFR that regulate cell survival in the presence of TNF. We used wortmannin and U0126 to inhibit AKT and ERK1/2, respectively. Wortmannin or U0126 alone did not induce apoptosis of BEAS-2B cells. Wortmannin significantly enhanced TNF-induced apoptosis, while U0126 did not affect TNF-induced apoptosis (Figure 5d,e). Therefore, the PI3K/AKT pathway may contribute to cell survival via TNF-induced EGFR transactivation. Although it would be difficult to recapitulate the SPC-TNF tg mouse model using BEAS-2B cells, we speculate that P38MAPK signal contributes to TNF-induced apoptosis [25]. 

### 2.6. EGFR Ligands, HB-EGF, and TGF-α Dominantly Regulate TNF-Induced EGFR Transactivation via TACE in BEAS-2B Cells

**Figure 6:**
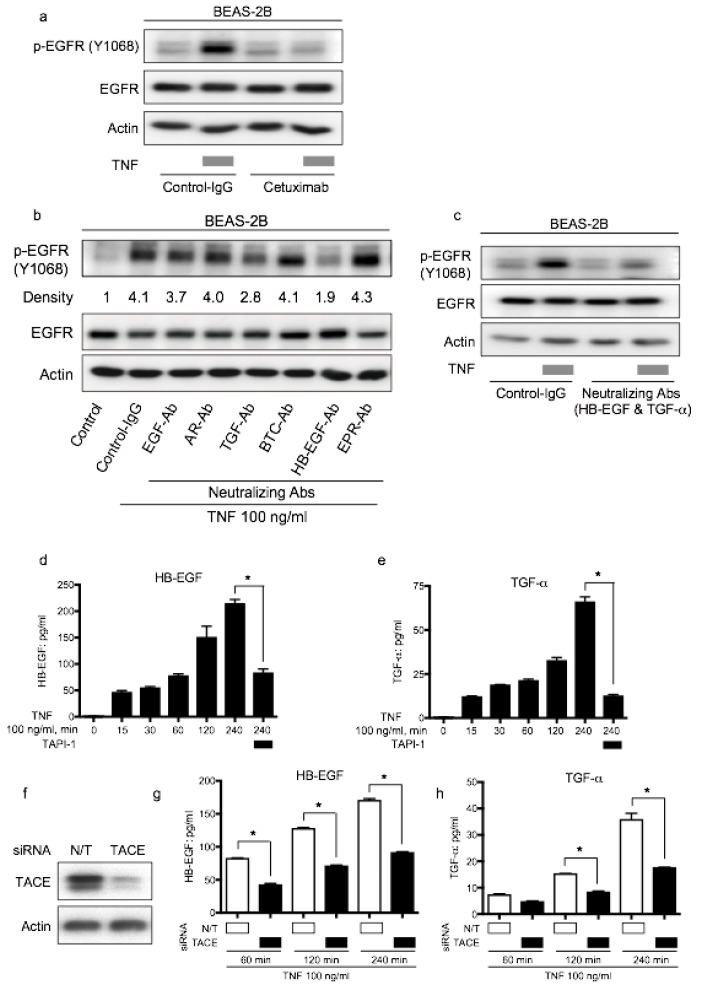
Epithelial growth factor receptor (EGFR) ligands, transforming growth factor (TGF)-α and heparin binding (HB)-EGF, promote tumor necrosis factor (TNF)-induced EGFR transactivation via the TNF-α converting enzyme (TACE). Lung epithelial cells (BEAS-2B) were cultured and serum-starved for 16 h prior to different treatments (**a–e**). Cell lysates were immunoblotted, and β-actin was included as a loading control (**a–c**). (**a**) BEAS-2B cells were treated with TNF (100 ng/mL) for 15 min following pretreatment with control antibody (control-IgG) or anti-EGFR antibody cetuximab (10 µg/mL) for 1 h. (**b**) BEAS-2B cells were treated with TNF (100 ng/mL) for 15 min with or without pretreatment (1 h) with neutralizing antibodies (10 µg/mL) for various kinds of EGFR ligands: EGF, amphiregulin (AR), TGF-β(TGF), β-cellulin (BTC), HB-EGF, and epiregulin (EPR). Band intensity of phosphorylated EGFR (P-EGFR) was quantified and determined using the ImageJ software (NIH). The relative ratios compared to the control are shown. (**c**) BEAS-2B cells were treated with TNF (100 ng/mL) for 15 min with or without pretreatment (1 h) with neutralizing antibodies against TGF-α (10 µg/mL) and HB-EGF (10 µg/mL). (**d,e**) BEAS-2B cells were treated with TNF (100 ng/mL) for the indicated duration in the presence or absence of the TACE inhibitor TAPI-1 (1 µM). (**d**) HB-EGF and (**e)** TGF-α protein levels in the culture supernatants were measured by enzyme-linked immunosorbent assay (ELISA). (**f,g**) BEAS-2B cells were cultured on collagen-coated dishes and transfected with non-targeting (N/T) small interfering RNA (siRNA) or TACE for 48 h. The cells were serum-starved for 16 h, and then treated with TNF (100 ng/mL) for 60, 120, and 240 min. The protein levels of (**f**) HB-EGF and (**g**) TGF-α in the culture supernatants were measured by ELISA. (**h**) BEAS-2B cells were cultured on collagen-coated dishes and transfected with N/T siRNA or TACE for 72 h. The cell lysates were collected and protein levels of TACE were determined by Western blot analysis.

Cetuximab is an EGFR-neutralizing antibody. It binds EGFR and downregulates ligand-induced EGFR phosphorylation [26]. To verify the involvement of EGFR ligands in TNF-induced EGFR transactivation, we used neutralizing antibodies for EGFR. Therefore, TNF-induced EGFR phosphorylation was inhibited by cetuximab, indicating that EGFR ligands contributed to EGFR transactivation by TNF (Figure 6a). To identify the EGFR ligands involved in TNF-induced EGFR phosphorylation, neutralizing antibodies were employed. As shown in Figure 6b, EGFR-transactivation by TNF was attenuated by the treatment of neutralizing antibodies against HB-EGF and TGF-α. Furthermore, the combination of neutralizing antibodies against HB-EGF and TGF-α exhibited remarkable inhibition of EGFR phosphorylation in the presence of TNF (Figure 6c). To measure the expression of HB-EGF and TGF-α in the culture supernatant, ELISA was performed. HB-EGF and TGF-α expression in the culture supernatant increased upon TNF exposure; however, the presence of TAPI-1 reduced the expression of these EGFR-ligands (Figure 6d,e). Moreover, knockdown of TACE using siRNA (Figure 6f), reduced the amount of HB-EGF and TGF-α in the culture supernatant (Figure 6g,h). These results indicate that the expression of HB-EGF and TGF-α in the culture supernatant was regulated by TACE and that these ligands were involved in TNF-induced transactivation in BEAS-2B cells. Taken together, lung epithelial cells BEAS-2B should be protected from TNF-induced apoptosis through EGFR transactivation via the autocrine loop of the EGFR ligands, HB-EGF and TGF-α, which are cleaved by TACE. 

## 3. Discussion

This study was performed to verify the hypothesis that EGFR-TKIs induce pneumonitis by blocking EGFR transactivation by TNF. The mechanisms of EGFR-TKI-induced pneumonitis have not been completely elucidated. However, this study provides evidence that EGFR tyrosine kinase activity prevents lung injury in a TNF-enriched environment. Furthermore, we have found that in lung epithelial cells BEAS-2B, TACE is required for TNF-induced EGFR activation and that PI3K/AKT is a target of EGFR transactivation. These findings show that transactivation of EGFR prevents TNF-induced lung epithelial cell apoptosis.

TNF-induced EGFR phosphorylation regulates critical physiological responses in acute lung injury. Airway inflammation, characterized by the release of the pro-inflammatory cytokine TNF, induces excessive EGFR signaling [27,28]. High amounts of EGFR ligands, amphiregulin and TGF-α secreted by bronchial epithelial cells result in the activation of EGFR in an autocrine loop, leading to increased mucus production by goblet cells [29,30]. Therefore, inhibiting EGFR tyrosine kinase activity may be beneficial to the treatment of hypersecretory diseases, such as asthma, chronic obstructive pulmonary disease (COPD), and bronchiectasis. Conversely, EGFR-ligands EGF or TGF-α can regulate lung epithelial cell repair in vitro and in vivo [31,32]. EGFR is overexpressed and activated in response to lung epithelial injury and play an important role in lung epithelial repair [33]. Increased amounts of both TGF-α and EGFR have been observed in bleomycin-injured lungs [34]. In addition, TGF-α has been identified in bronchoalveolar lavage fluid from patients with acute lung injury [34,35]. Taken together, the expression of EGFR or EGFR-ligands, and EGFR tyrosine kinase activity protect lung epithelial cells from apoptosis and enhance their recovery from lung inflammation and injury [36]. However, further studies are required to discover the role of EGFR and its ligands in inflammatory lung diseases. In this study, EGFR tyrosine kinase was transactivated by TNF in SPC-TNF tg mice. Inhibition of EGFR tyrosine kinase by gefitinib increased TNF-induced apoptosis in epithelial cells and significantly enhanced lung injury. The results of this study are consistent with those of previous reports, which showed that activation of EGFR and other ErbB family members protects colon epithelial cells from TNF-induced apoptosis [20,37].

For understanding the development of pneumonitis in whole lung, it is essential to evaluate lung inflammation by EGFR-TKI gefitinib administration ex vivo. In regions of inflammation in arthritis and tumors, vascular endothelial cells are activated by inflammatory cytokines, such as TGF-α and TNF. Leukocytes roll on the vascular endothelial cells by binding E-selectin on the vascular endothelial cell surface. Leukocytes infiltrate the vascular endothelium, and then migrate and accumulate in the inflammatory region. The sugar chain sialyl Lewis X (SLX) is well characterized as an E-selectin ligand, expressed on the surface of leukocytes. Therefore, SLX-liposomes primarily targeting E-selectin in activated endothelial cells deliver and accumulate their ICG content in regions of inflammation and within tumors [38,39]. Therefore, we employed an SLX-Lipo-ICG to evaluate lung tissue inflammation after administration of gefitinib in WT and SPC-TNF tg mice. The basal accumulation of SLX-Lipo-ICG in SPC-TNF tg mice was higher than in WT mice. Furthermore, after gefitinib administration, the accumulation of SLX-Lipo-ICG was enhanced in SPC-TNF tg mice but not in WT mice. These results indicate that gefitinib remarkably enhances TNF-induced lung inflammation in SPC-TNF tg mice.

Based on our results, IL-17A was the most significantly increased cytokine after gefitinib treatment in SPC-TNF tg mice. IL-17A appears to act primarily on non-hematopoietic cells, such as endothelial cells, epithelial cells, and fibroblasts [40]. Increased expression of IL-17A has been linked to inflammatory diseases such as asthma, cystic fibrosis, and COPD in the airway, as well as inflammatory bowel disease, and it appears to play an important protective role against infection [41,42,43]. Iyoda et al. reported that IL-17A and IL-17F can stimulate the phosphorylation of p38 MAPK and then enhance the expression of cytokines such as monocyte chemoattractant protein (MCP)-1 and macrophage inflammatory protein (MIP)-2 in cultured mouse mesangial cells [44]. In the SPC-TNF tg mice, gefitinib treatment led to increased phosphorylation of p38 MAPK, suggesting that IL-17A may stimulate p38 MAPK activity and induce lung epithelial cell apoptosis.

A meta-analysis of 16 trials on EGFR TKI treatment in lung cancer patients revealed that pneumonitis was the most common cause of death related to EGFR TKI toxicity [4]. A previous clinical study has identified risk factors of EGFR TKI-induced pneumonitis including old age, smoking history, pre-existing interstitial lung disease, and poor performance status [45]. For example, higher plasma concentration of TNF was measured in the older group than in the younger group [46]. Smoking may influence TNF-mediated systemic inflammation, such as in COPD or interstitial pneumonitis [47]. Furthermore, poor-performance status and cachexia, associated with cytokines of IL-6 and TNF, are well known [48]. Taken together, these risk factors are associated with TNF-enriched lung conditions. Therefore, for cases of EGFR TKI-induced pneumonitis, EGFR tyrosine kinase activity may be required to maintain homeostasis in TNF-rich lung environments where chronic inflammation is present.

In summary, we were able to elucidate the molecular mechanisms related to EGFR-TKI-induced lung injury using SPC-TNF tg mice. EGFR activation prevents TNF-induced apoptosis and severe inflammation. IL-17A was the most upregulated cytokine and may be involved. Furthermore, in lung epithelial cells BEAS-2B, we found that TNF activates EGFR phosphorylation by stimulating TGF-α and HB-EGF via TACE activity. This EGFR transactivation by TNF regulates cell survival. Taken together, our findings provide the biological mechanisms underlying EGFR-TKI-induced lung injury. Therefore, our findings suggest that caution will have to be taken prior to administration of EGFR-TKI in NSCLC cases that are associated with high levels of TNF or inflammation. This study has potential limitations. The effects reported are based on SPC-TNF tg mice, which highly express TNF in whole lung. We cannot, however, exclude the effect of other cytokines and signal transductions associated with lung fibrosis; these would be addressed in future research. 

## 4. Materials and Methods

### 4.1. Growth Factors, Antibodies and Inhibitors 

Human recombinant EGF and human recombinant TNF were purchased from Peprotech (Rocky Hill, NJ, USA). Gefitinib was kindly provided by AstraZeneca (Tokyo, Japan). TAPI-1 was from Calbiochem (San Diego, CA, USA). The following antibodies were purchased from Cell Signaling Technology (Danvers, MA, USA): Total EGFR antibody (#4267), phospho-EGFR (Y1068) antibody (#3777), total AKT antibody (#9272), phospho-AKT (S473) antibody (#9271), total ERK1/2 antibody (#9102), phospho-ERK1/2 antibody (#4370), phospho-MKK3/6 antibody (#9236), total p38 MAPK antibody (#9212), phospho-p38 MAPK antibody (#9211), TACE antibody (#3976), and β-actin antibody (#4970). Meanwhile, antibodies specific to total MKK 3/6 (sc-13069) was purchased from Santa Cruz Biotechnology, Dallas, TX, USA. Cetuximab was purchased from Showa University Hospital (Shinagawa, Tokyo, Japan). Materials used for western blot analysis were from Bio-Rad Laboratories (Richmond, CA, USA). Dulbecco’s modified Eagle medium (DMEM) and all other materials were purchased from Sigma Chemical (St. Louis, MO, USA) unless otherwise stated. All pharmacological inhibitors and agonists were dissolved in DMSO and added to the medium with a final DMSO concentration of <0.1%.

### 4.2. Cell Culture

BEAS-2B cells (ATCC CRL-9609™) were from the American Type Culture Collection (ATCC^®^). Cells were cultured in collagen-coated dishes and maintained in DMEM with 10% fetal bovine serum (FBS) and 100 U/mL penicillin and streptomycin at 37 °C with 5% CO_2_. Before experiments, cells were transferred to DMEM with 0.1% FBS and 100 U/mL penicillin and streptomycin at 37 °C with 5% CO_2_ for 16−18 h.

### 4.3. Transient Transfection of siRNA 

BEAS-2B cells (70% confluence) were transiently transfected with SMARTpool siRNA (Dharmacon, Lafayette, CO) that were non-targeting (N/T), or against human EGFR or human TACE using Lipofectamine 2000 according to the manufacturer’s instructions. After 24 h of transfection, cells were cultured in serum-starved medium for 16–18 h before treatment.

### 4.4. Cell Lysate Preparation and Western Blot Analysis 

Cells were washed twice with ice-cold PBS and scraped into the cell lysis buffer (1% Nonidet P-40; 120 mM NaCl; 50 mM Tris-HCl, pH 7.4; and 1% protease and phosphatase II & III inhibitor cocktail). Lysates were incubated on ice for 20 min and clarified by centrifugation (14,000 rpm, 10 min). Protein content in the supernatant was determined by the bicinchoninic acid (BCA) method. Equivalent amounts of protein were mixed with Laemmli sample buffer, and after incubation at 95 °C for 5 min, the samples were separated by 8–12% sodium dodecyl sulfate polyacrylamide gel electrophoresis (SDS-PAGE). Gels were transferred to a polyvinylidene fluoride membrane and then blocked with 5% nonfat dry milk in Tris-buffered saline (TBS: 50 mM Tris, 150 mM NaCl, 25 mM KCl, pH 8.0) with 0.05% Tween 20 (TBST) for 1 h at room temperature. Membranes were then exposed to the primary antibodies overnight at 4 °C following the manufacturer’s suggested dilutions (1:1000 dilution). Membranes were washed with TBST, exposed to the secondary antibody (1:2000 dilution) for 1 h in TBST, and washed in TBST. Immunoreactive bands were visualized with the Western Lightning Enhanced Chemiluminescence kit (PerkinElmer Life Sciences, Waltham, MA, USA). Densitometric analysis was performed using the ImageJ software (NIH). 

### 4.5. Real-Time RT-PCR

cDNA was synthesized from total RNA isolated from whole lung samples and analyzed by real time PCR using Powerup SYBR Green mastermix (Applied Biosystems) and a fluorexcence-based RT-PCR-detection system (GeneAmp 5700; Applied Biosystems). Specific primer sets are shown in supplementary Table S1. *GAPDH* was used as an internal control. All PCR reactions were done in duplicate for both the target gene and internal control. The relative gene expressions were calculated according to the 2-ΔΔCt method, and the control values were expressed as 1 [49]. 

### 4.6. Mice, EGFR-TKIs Administration, and Tissue Preparations 

All animal experiments were performed according to protocols approved by the Institutional Animal Care and Use Committee at Showa University (code; 57003, date; April 1st, 2017). SPC-TNF transgenic mice were obtained from Masaki Fujita (Fukuoka University, Fukuoka, Japan) on a C57BL/6 background. PCR primers specific to the TNF sequence were used for genotyping as described by Miyazaki et al. [21]. Mice (*n* = 8 per group) (6-8 weeks old, 20−25 g) were once daily orally administered with gefitinib (100 mg/kg or 200 mg/kg in H_2_O containing 1% tween 80) or with 1% tween 80 in H_2_O as control for 14 days. Lung tissue was fixed in 20% neutral-buffered formalin and was paraffin-embedded before sectioning. Lung tissue was ground in homogenization buffer and lysed for protein analysis.

### 4.7. Apoptosis Assay

Apoptosis was detected in lung sections by ApopTag Peroxidase In Situ Oligo Ligation Kit staining using T4 DNA ligase following the manufacturer’s guidelines (Merck Millipore, Billerica, MA). Results were observed by differential interference contrast microscopy with relative apoptosis determined from at least 500 cells. ApopTag Fluorescein In Situ Apoptosis Detection Kit (TUNEL) was used, and 4′,6-diaminido-2-phenylindole (DAPI) staining was performed as described previously [20]. The slides were observed by fluorescence microscopy, and percent apoptosis was determined from at least 500 cells.

### 4.8. Enzyme-Linked Immunosorbent Assay (ELISA)

Human HB-EGF and human TGF-α in culture supernatant were measured by ELISA (R&D Systems) according to manufacturer’s instructions. To prevent binding of EGF ligands with EGFR, TACE inhibitor, TAPI-1 (1 µM) was pretreated for 60 min, and then were treated with TNF (100 ng/mL) in various durations. Supernatants were analyzed by ELISA according to manufacturer’s recommendations.

### 4.9. Statistical Analysis 

Significant difference between two groups was determined by paired Student’s *t*-test, with statistical significance set to *p* < 0.05. Each experiment was repeated at least three times on separate occasions.

## Figures and Tables

**Table 1:** Cytokine mRNA expression profile of wildtype (WT) vs surfactant protein C-tumor necrosis factor transgenic (SPC-TNF tg) mouse lung tissue. Lung mRNA transcripts of WT and SPC-TNF tg mice were quantified by real-time PCR and normalized to *GAPDH*. The data are represented relative to control values, as means ± SEM (*n* = 8). Significant values are indicated in boldface.

	Wild Type	SPC-TNF tg	*p*
*Ifng*	1	0.9 ± 0.1	0.53
*Il1b*	1	2.1 ± 0.4	**0.011**
*Il6*	1	0.5 ± 0.0	**<0.0001**
*Il10*	1	2.6 ± 0.2	**0.0004**
*Il17a*	1	2.6 ± 0.4	**0.0088**
*Il21*	1	0.6 ± 0.1	**0.0015**
*Il23a*	1	1.9 ± 0.3	**0.01**
*Tnf*	1	825.3 ± 110	**0.0002**
*Tgfb1*	1	1.6 ± 0.4	**<0.0001**
*Foxp3*	1	5.1 ± 0.6	**0.0003**
*Gata3*	1	0.6 ± 0.0	**<0.0001**
*Tbet*	1	0.8 ± 0.1	0.37
*Gm-csf*	1	0.7 ± 0.1	0.11
*Egfr*	1	0.8 ± 0.2	0.06

**Table 2:** Cytokine mRNA expression profile of wild type (WT) mouse lung tissue with gefitinib 100 mg/kg and 200 mg/kg for 14 days. The lung tissue mRNA transcripts were quantified by real-time PCR and normalized to *GAPDH*. The lung tissue mRNA transcripts were quantified by real-time PCR and normalized to *GAPDH*. The data are represented relative to control values, as means ± SEM (*n* = 8). Significant values are indicated in boldface.

	Control	Gef 100 mg/kg	*p*	Gef 200 mg/kg	*p*
*Ifng*	1	0.89 ± 0.1	0.37	0.69 ± 0.2	0.086
*Il1b*	1	1.9 ± 0.1	**<0.0001**	13.7 ± 2.3	**0.0008**
*Il6*	1	1.1 ± 0.1	0.31	1.7 ± 0.1	**<0.0001**
*Il10*	1	1.3 ± 0.1	**0.0093**	1.7 ± 0.1	**0.0005**
*Il17a*	1	1.0 ± 0.1	0.93	2.6 ± 0.5	**0.024**
*Il21*	1	1.1 ± 0.1	0.27	1.1 ± 0.1	0.15
*Il23a*	1	1.1 ± 0.0	0.086	1.0 ± 0.0	0.86
*Tnf*	1	1.4 ± 0.0	0.17	1.4 ± 0.2	0.093
*Tgfb1*	1	0.93 ± 0.0	0.076	0.97 ± 0.0	0.34
*Foxp3*	1	1.6 ± 0.1	**<0.0001**	1.6 ± 0.1	**0.0051**
*Gata3*	1	0.99 ± 0.1	0.97	0.6 ± 0.1	**0.0015**
*Tbet*	1	0.82 ± 0.1	**0.017**	0.5 ± 0.1	**0.0012**
*Gm-csf*	1	0.80 ± 0.1	0.057	0.8 ± 0.1	0.16
*Egfr*	1	1.0 ± 0.1	0.94	1.0 ± 0.2	0.87

**Table 3:** Cytokine mRNA expression profile of surfactant protein C-tumor necrosis factor transgenic (SPC-TNF tg) mouse lung tissue with gefitinib 100 mg/kg and 200 mg/kg for 14 days. The lung tissue mRNA transcripts were quantified by real-time PCR and normalized to *GAPDH*. Data are represented relative to control values, as mean ± SEM (*n* = 8). Significant values are indicated in boldface.

	Control	Gef 100 mg/kg	*p*	Gef 200 mg/kg	*p*
*Ifng*	1	0.8 ± 0.0	**0.0004**	0.8 ± 0.1	**0.034**
*Il1b*	1	0.8 ± 0.1	0.095	1.2 ± 0.1	**0.0011**
*Il6*	1	1.3 ± 0.0	**<0.0001**	1.9 ± 0.2	**0.001**
*Il10*	1	1.0 ± 0.1	0.96	1.6 ± 0.1	**0.0003**
*Il17a*	1	1.5 ± 0.1	**0.0018**	5.8 ± 0.4	**0.014**
*Il21*	1	1.3 ± 0.1	**0.027**	1.5 ± 0.1	**0.0046**
*Il23a*	1	0.9 ± 0.1	0.16	1.1 ± 0.1	0.11
*Tnf*	1	0.9 ± 0.0	**0.0057**	1.2 ± 0.1	**0.0001**
*Tgfb1*	1	1.2 ± 0.1	**0.0042**	1.9 ± 0.2	**0.003**
*Foxp3*	1	0.9 ± 0.0	0.24	1.1 ± 0.2	**0.042**
*Gata3*	1	1.0 ± 0.1	0.69	0.8 ± 0.0	**<0.0001**
*Tbet*	1	1.1 ± 0.0	0.32	1.2 ± 0.2	0.49
*Gm-csf*	1	0.7 ± 0.0	**<0.0001**	1.1 ± 0.1	0.22
*Egfr*	1	1.1 ± 0.1	0.53	1.1 ± 0.0	0.14

## Supplementary Materials

Supplementary materials can be found at https://www.mdpi.com/1422-0067/20/16/4021/s1.
